# Surface hardening of a mould steel by laser quenching

**DOI:** 10.1038/s41598-026-42194-8

**Published:** 2026-03-10

**Authors:** F. M. Rodrigues, F. Gonçalves, D. Cavaleiro, E. L. Silva, A. S. Ramos

**Affiliations:** 1 CEMMPRE - Department of Mechanical Engineering, University of Coimbra, R. Luís Reis Santos, 3030-788 Coimbra, Portugal; 2 Durit Coatings, Parque Industrial de Taveiro nº 41 e 42, Taveiro, 3045-508 Coimbra, Portugal

**Keywords:** Laser quenching, Mould steel, Nanoindentation, Wear resistance, Engineering, Materials science

## Abstract

The objective of this work is to study the laser quenching process of AISI P20 + S mould steel, focusing on improving surface hardness and wear resistance. AISI P20 + S steel was laser-quenched using temperatures of 1000 °C and 1200 °C, and laser beam overlap rates of 10% and 25%. The as-received and laser quenched steel was characterized by optical microscopy, scanning electron microscopy, and X-ray diffraction. After laser quenching, nanoindentation and pin-on-disk tests were performed to determine hardness profiles and assess wear resistance, respectively. The hardness at the surface more than doubled compared to the hardness of the untreated steel, confirming the formation of martensite. The 1000 °C laser quenching resulted in a depth lower than 700 μm, with a surface hardness close to 8 GPa. At 1200 °C, the depth increased to nearly 1400 μm, and the hardness reached 8–9 GPa, although the thicker oxide layer formed during the laser treatment led to an increase in the wear coefficient and volume. Due to the detachment of oxide layers, the main wear mechanism observed was adhesive. The work carried out confirmed the potential of laser quenching in the surface hardening of mould steels.

**Supplementary Information:**

The online version contains supplementary material available at 10.1038/s41598-026-42194-8.

## Introduction

One of the main advantages of steel as structural material is the potential to broaden the range of properties and performance through thermal, thermochemical or mechanical processes^1,2^. Surface hardening by quenching is one of the most important and widely used thermal processes, essential to improve the mechanical properties of steels, namely hardness and wear resistance^1^. This process involves heating the steel to a high temperature (austenization), followed by rapid cooling, which results in the formation of martensite. Martensite is a hard and brittle phase that is responsible for a high resistance to wear, making it ideal for applications where durability is crucial, such as cutting tools, injection moulds, and mechanical components subject to high stresses^3–5^. In addition, the transformation of austenite into martensite generates compressive stresses in the outer layer that hinder crack propagation.

Conventional quenching has some limitations, especially when applied to parts with complex geometries. The rapid cooling required to form martensite can cause distortions and residual stresses, compromising the parts’ dimensional accuracy and structural integrity^6^. Additionally, on surfaces with irregular shapes or intricate details, the conventional quenching process can be difficult to control. In this context, laser quenching stands out as an innovative solution^7,8^. This process consists of using a laser beam to selectively heat the surface of a steel part, allowing for rapid and controlled cooling by thermal conduction, without the need for external cooling media. The rapid cooling rates are high enough to exceed the CCT (Continuous Cooling Transformation) diagram nose, resulting in martensitic structures^9^. Laser hardening reduces distortions and residual stresses common in conventional methods, making it especially advantageous for parts with complex geometries^10^. Since the heating is very localized, the core of the part remains relatively cool and therefore maintains its ductility, while the hardened surface offers high wear resistance. In addition, the surface of the material usually does not require post-processing, as it remains rather smooth after laser treatment. Laser surface hardening is mainly applied to ferrous alloys such as stainless steels, tool steels and cast irons^11^. The hardening capacity depends on the laser processing parameters, namely laser power, wavelength, beam shape and area, scanning speed and focusing conditions, being therefore essential to optimize these parameters to improve the wear resistance and durability of the steels^12^. The percentage of overlap of the laser tracks can also influence the properties of the laser surface hardened steels. Several studies have been conducted to improve, through laser quenching, the properties of low and high alloy steels^13–15^. Moradi et al.^16^ used a high-power diode laser for the treatment of AISI 410 and AISI 420 stainless steels. This study revealed that the AISI 420 stainless steel has higher surface hardness and lower penetration depth and width than the AISI 410 stainless steel. Comparison of these results with those from furnace quenching showed that the laser surface hardening process is more effective and precise than conventional quenching^16^.

Laser quenching can be particularly promising for mould steels, designed to withstand millions of cycles during the mass production of plastic products. These tools are subject to intense thermomechanical loads that can impair surface quality and durability, impacting productivity. Surface damage occurring in moulds often results from wear, fatigue cracking, and corrosion. To mitigate these problems, thermal and thermochemical treatments have been explored to improve injection moulds’ surface. AISI P20 and P20 modified steels are widely used in the moulds industry, since they offer high resistance to thermal shock and abrasion. In this context, Park et al.^17,18^ studied the impact of hardness on the wear and corrosion behaviour of AISI P20-improved steel by using a high-power diode laser. The microstructure of the base metal changed from tempered martensite to lath-type martensite, and thus laser heat treatment increased the hardness of the steel, resulting in a significant improvement in wear resistance. Fretting tests showed that laser-treated samples had less volume and less wear marks compared to the base metal. Furthermore, the laser-treated samples exhibited more stable friction coefficient profiles and surfaces with few abrasion grooves^17^. However, the laser surface treatment showed a marginal influence on the corrosion resistance of the AISI P20-improved steel^18^. The effect of laser surface treatment varying parameters such as scanning speed, was analysed by Lagarinhos et al.^12^ in order to determine the best parameters to increase the surface hardness of modified P20 steel. Laser quenching resulted in an increase in hardness from about 3 to 6.1 GPa^12^. The heat treatment depth was approximately 1.0 mm, and the treated steels demonstrated a slightly better distribution and deeper hardness profiles for lower scanning speeds^12^.

Laser surface hardening has shown potential for improving the tribological properties and extending the service life of plastic injection moulds. However, studies focusing the impact of laser quenching on surface hardening of mould steels and the optimization of parameters in order to make the laser treatment more effective are still lacking.

The main objective of this research is to evaluate the potential of the laser hardening process of AISI P20 + S steel, in order to obtain surfaces with high hardness and, therefore, high wear resistance. To attain this goal, it is necessary to adjust the conditions of temperature, laser power, scanning speed and laser beam size, in order to maximize the formation of martensite on the surface without compromising the integrity of the core. The ultimate goal is to develop a robust and efficient process that can be applied industrially, ensuring parts with optimized mechanical properties and a long service life. Although laser quenching is an established technology, its application remains confined to the laboratory scale. By contrast, carburising and nitriding are widely adopted industrial processes for the surface hardening of steels. Within this context, the novelty of the present work lies in its contribution to increasing the Technology Readiness Level (TRL) of laser hardening from laboratory validation (TRL 4) towards a fully deployable industrial process. This advancement will be demonstrated through laser surface hardening of steels employed in case studies defined by mould and automotive industries participating in the project under which this work is carried out.

## Experimental details

### Materials

A low-alloy tool steel was selected to study the influence of laser quenching. This steel is classified as: AISI P20 + S, DIN 1.2312, ASTM A681. P20 + S steel is commonly used in the manufacturing of tools for injection moulds. Its nominal chemical composition, in weight%, is presented in Table 1, together with Si, Mn and Cr contents measured by energy dispersive spectroscopy (EDS).

**Table 1:** Chemical composition of the P20 + S mould steel (wt%).

Steel	C	Si	Mn	P	S	Cr	Mo
P20 + S	0.35–0.45	0.30–0.50 0.4–0.5*	1.40–1.60 1.2–1.5*	≤ 0.03	0.05–0.10	1.80–2.10 1.6–1.8*	0.15–0.25

*Current work (measured by EDS).

For comparison purposes, the as-received steel was characterized after preparation using standard metallographic procedures, including grinding using silicon carbide papers up to 2500 mesh and polishing with 3 μm diamond suspension. This polishing provided a uniform mirror-finish surface, suitable for subsequent characterization. Etching using Nital reagent revealed the steel microstructure.

### Laser quenching

Laser quenching was performed using a Laserline diode laser (LDF 4000-60 model) with a power of 4 kW and a wavelength ranging from 980 to 1020 nm. To ensure uniform and efficient application of the beam over the steel samples, the apparatus was equipped with OTZ lenses, which contributed to better precision and distribution of the laser beam.

During the laser quenching process, the laser beam dimensions were fixed at 8 × 8 mm, and the scanning speed was kept constant (200 mm/min), while the temperature and the overlap rate were varied. To analyse the effect of different heat levels, two different temperatures were used for the laser treatment: T1 = 1000 °C and T2 = 1200 °C. The temperature was monitored and controlled in real time by a two colour pyrometer with an accuracy of ±1.5 °C, which automatically adjusted the laser power (880–1144 W) to ensure the surface remained within the desired temperature. Regarding the overlap rate (OR), for each temperature four tracks were made using two different overlap percentages: OR1 = 10% and OR2 = 25%, which resulted in an overlap between consecutive tracks of 0.8 and 2 mm, respectively. The different overlap rates aimed to evaluate how the extent of the overlap regions interferes with the heat treatment.

For each combination Temperature/Overlap rate, three steel samples were treated by laser quenching. The steel samples for laser quenching were prepared by grinding using silicon carbide papers up to 1200 mesh. After quenching, some of the samples were cut, mounted in resin, and their cross-section prepared by conventional grinding and polishing. For the laser-treated samples, the following nomenclature was adopted: P20_Temperature_Overlap rate.

### Characterization techniques

#### (Micro)structural characterization

The initial observation of the microstructure of the as-received and treated steel was carried by optical microscopy, using a Leica microscope, model DM4000, equipped with a photographic camera. More detailed microstructural analysis and morphological characterization was performed by scanning electron microscopy (SEM), in secondary electrons (SE) and backscattered electrons (BSE) mode. A Hitachi SEM, model SU3800, was used using accelerating voltages of 10 or 15 kV. This microscope is equipped with EDS, which allowed the chemical composition of the steel samples to be evaluated.

The crystalline phases present in the as-received and treated steel were identified by x-ray diffraction (XRD). XRD analyses were carried out using a Rigaku diffractometer, Smartlab model, coupled to a PW 3020/00 goniometer, with Cu Kα radiation (λ = 1.54060 Å). XRD experiments were performed at 40 kV and 50 mA, using an angular range between 30 < 2θ < 100°, with 0.025° spacing.

#### Mechanical and tribological characterization

Nanoindentation was used to evaluate the mechanical properties, namely hardness and Young’s modulus. Due to the reduced loads used, nanoindentation permits spacings of a few micrometers between consecutive tests, allowing detailed hardness profiles to be obtained along the steel samples treated by laser quenching.

Mechanical characterization was accomplished using a MicroMaterials nanoindenter, model NanoTest. The nanoindenter is equipped with a Berkovich indenter and the area function was previously calibrated using a fused silica sample. The depth-load curves were acquired by applying load for 30 s until attaining a maximum load of 150 mN, which was maintained for 30 s, following load relieve until reaching approximately 10% of the maximum load and maintenance for another 30 s. The second creep, at a load close to 15 mN, allows for thermal drift correction. Nanoindentation results were treated using the method of Oliver and Pharr^19^.

**Fig. 1:**
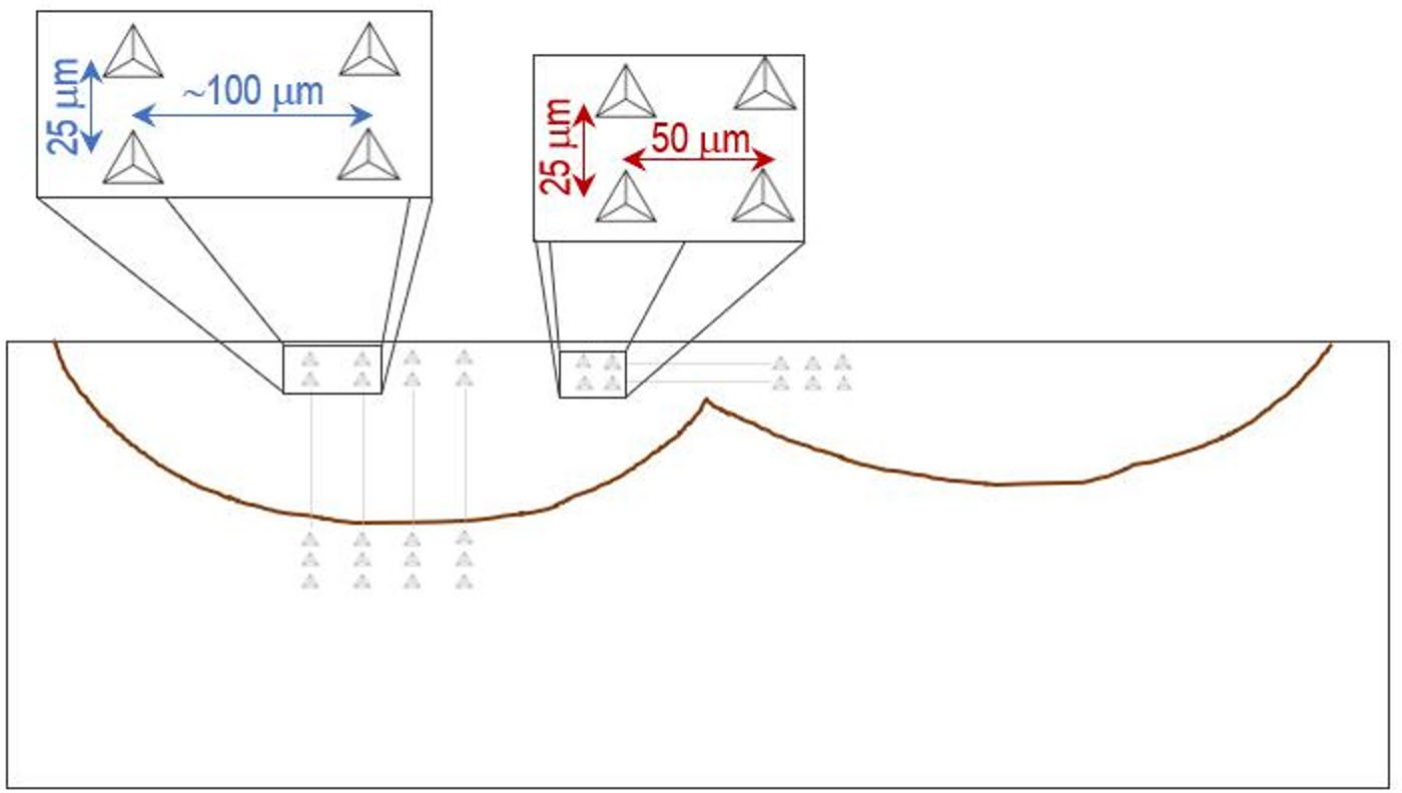
Scheme of the nanoindentation tests on sample P20_1200_25. Experiments for depth profiles (right) and across overlap zone (centre).

For the laser quenched steel, 3–6 profiles were made per sample with 25 μm spacings between indentations. Each profile started approximately 25 μm below the sample surface and completed after performing some tests on the untreated steel. Depending on the depth of the treated areas, between 30 and 70 tests were carried out per profile. To evaluate the influence of laser overlap, two additional profiles parallel to the surface and across the overlap zone were made for sample P20_1200_25 (Fig. 1).

The wear behaviour was evaluated by means of pin-on-disk tests (CSM Instruments) performed in a dry environment, with relative humidity between 40 and 50%, following ASTM standard^20^. Silicon nitride (Si_3_N_4_) spheres (10 mm in diameter) were used as counterbody. In a preliminary test, a load of 5 N was applied and the number of cycles was set to 5000. However, due to the absence of wear, the conditions were adjusted. The load was increased to 10 N, with a linear speed of 0.1 m/s, during 5000 cycles. For each combination of temperature and overlap rate, two tests were performed. Before each test, the tribological pair (sphere and disk) was meticulously cleaned with alcohol.

After the pin-on-disk tests, the wear tracks were examined by SEM and profilometry. Dimensional variations were used to calculate the wear volume and coefficient. The grooves formed on the wear tracks were analyzed with a Mitutoyo surftest SJ-500 profilometer, which allows the depth and width of the grooves to be measured. With these data, it is possible to determine the specific wear rate, *k*, which is normalized in relation to the applied normal load and expressed by the formula^21^:

$$k=\frac{V}{N\times L}$$

where V is the volume of material removed (mm^3^), N is the sliding distance ran during the test (m), and L is the applied normal load (N). The volume of material removed was estimated based on the cross-sectional area of the grooves or craters calculated from three randomly zones on each track.

## Results and discussion

### As-received Steel

**Fig. 2:**
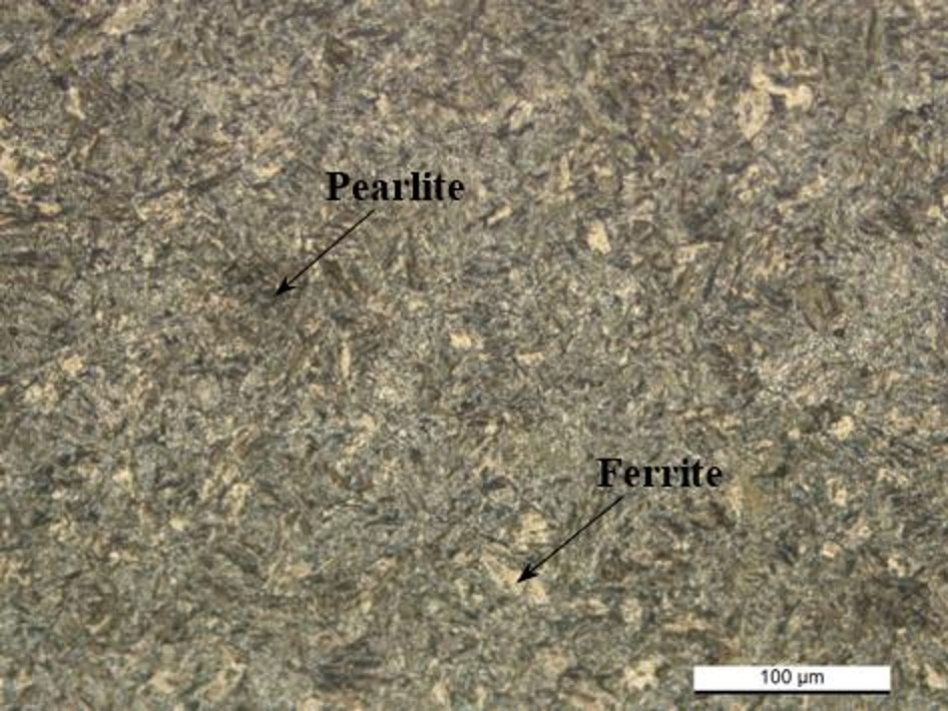
OM image of the P20 + S steel chemically etched.

Figure 2 shows optical microscopy (OM) images of the as-received low alloy steel. Microstructure analysis reveals that the steel is composed of ferrite and pearlite. Ferrite (lighter grains) contributes to the toughness of the steel, while pearlite (darker lamellar grains) increases the hardness. The phase distribution is relatively uniform, and the images show a fine-grained microstructure characteristic of P20 steel.

Nanoindentation tests were carried out using the experimental conditions selected for the hardness profiles of the laser quenched samples. According to the results, the P20 + S steel has a hardness of 3.4 ± 0.22 GPa, close to the value indicated in the steel technical data sheet.

### Laser quenched steel

#### (Micro)structural characterization

**Fig. 3:**
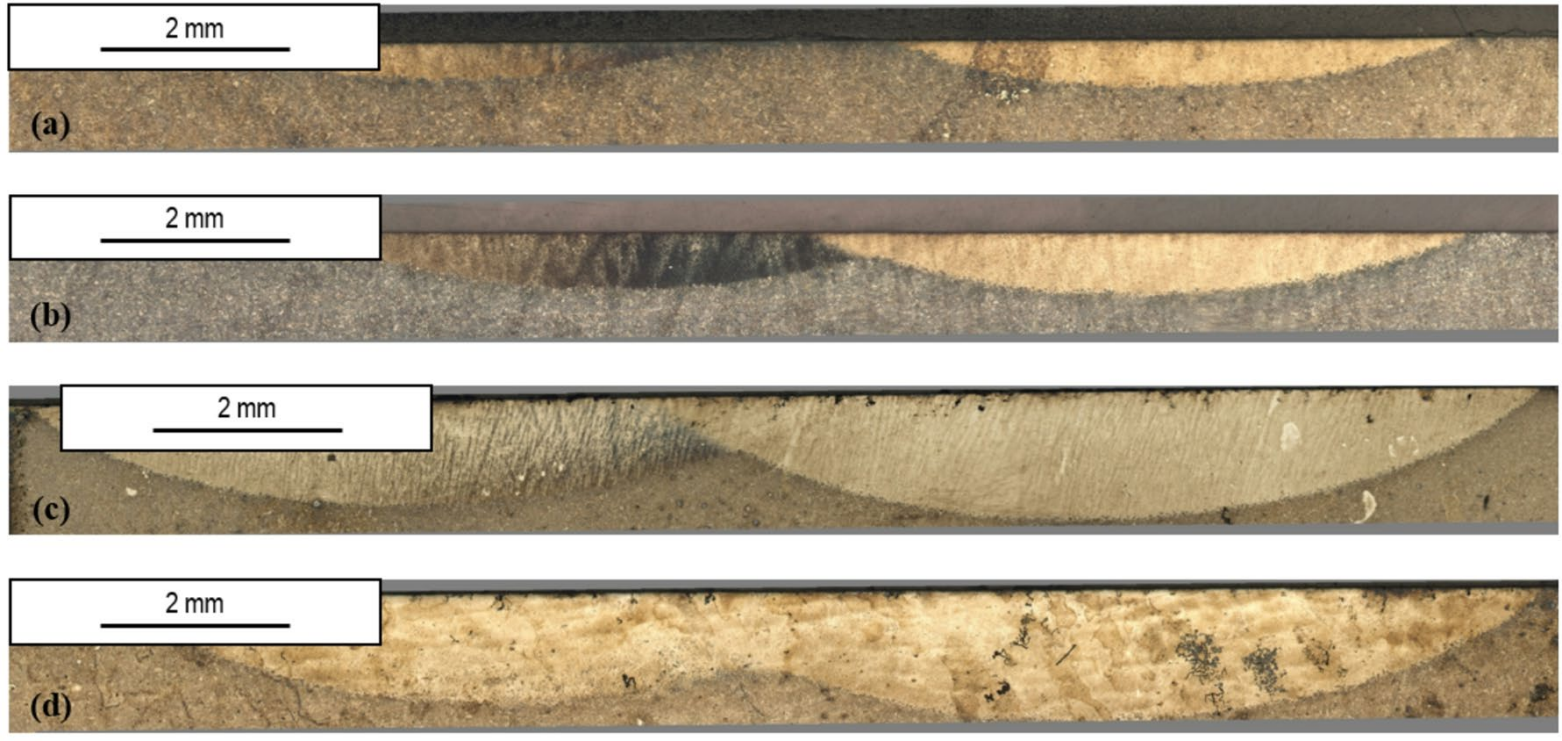
OM images of the laser quenched steel. (**a**) P20_1000C_10%; (**b**) P20_1000C_25%; (**c**) P20_1200C_10%; (**d**) P20_1200C_25%.

The laser quenched samples were carefully analysed by OM (Fig. 3). The analysis revealed significant differences of the laser treated samples using different temperatures and overlap rates.

In sample P20_1000C_10%, the marks resulting from the two laser tracks are visible, without observing in Fig. 3a overlap of the tracks. Keeping the temperature at 1000 °C and increasing the overlap rate to 25%, two partially overlapped marks are visible (Fig. 3b). The maximum depth is slightly higher than that observed using 10% overlap, suggesting that the higher overlap rate favours tracks’ depth.

The P20_1200C_10% sample, shown in Fig. 3c, stands out by exhibiting clear quench marks with noticeable overlap, even for an overlap rate of 10% (Fig. 3c), with a significant increase in maximum depth in relation to the samples heated up to 1000 °C. Finally, sample P20_1200C_25%, which was subjected to the same heat treatment but with an overlap rate of 25%, also shows clear signs of quenching, with obvious overlap of the two marks (Fig. 3d).

**Fig. 4:**
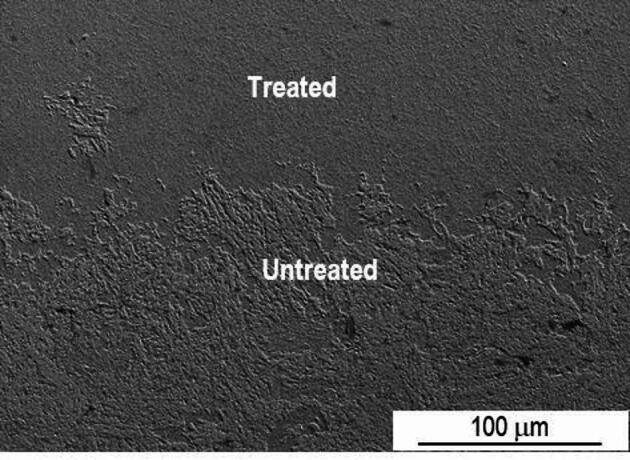
SEM SE image of the interface between the treated and untreated zone of sample P20_1000C_10%.

To examine the microstructure modifications resulting from the laser treatment, SEM analysis were carried out. The laser mark of sample P20_1000C_10% shown in Fig. 4 is about 500 μm deep, with the interface between the treated and untreated zones clearly visible. As observed by OM, it was not possible to identify the zone corresponding to the overlap of the two laser tracks.

**Fig. 5:**
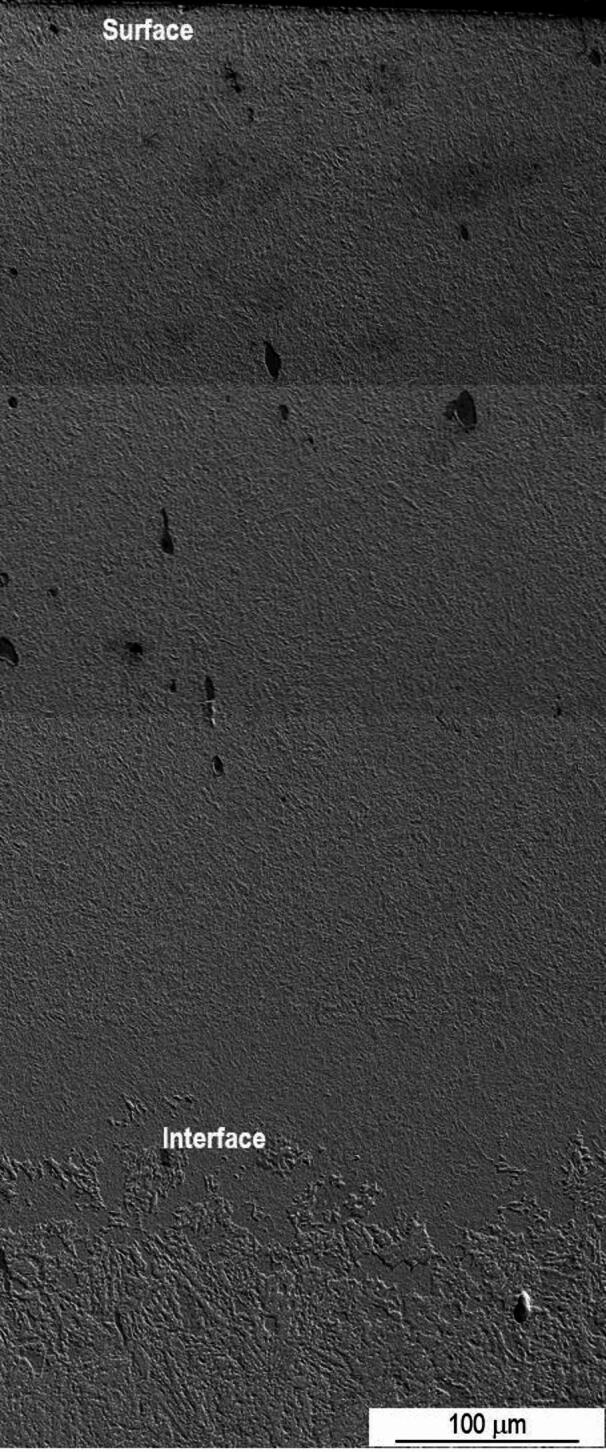
Reconstruction of the laser treated zone of sample P20_1000C_25%.

**Fig. 6:**
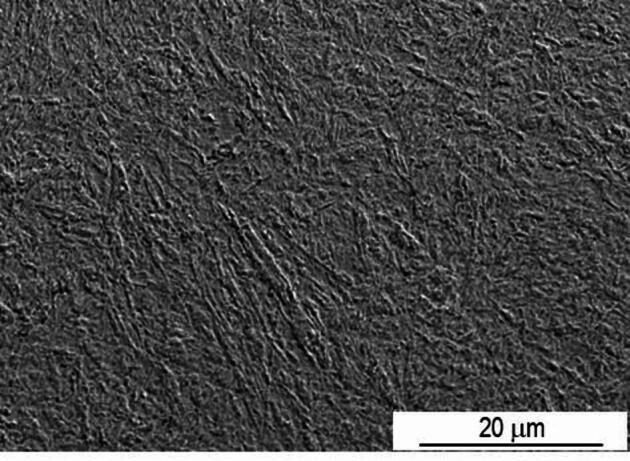
SEM SE image showing the microstructure of sample P20_1000C_25% close to the treated surface.

Regarding sample P20_1000C_25%, the interface between the treated and untreated zones is also clearly visible, and the deepest track has approximately 700 μm. Figure 5 shows the corresponding reconstruction of the area affected by the laser treatment. The microstructure close to the surface (quenched zone) is visible in more detail in Fig. 6. As can be seen, due to the martensitic transformation promoted by laser quenching, the microstructure is distinct from that of the untreated steel. The typical microstructure of a hypoeutectoid steel disappears after laser treatment.

Despite the higher overlap rate, in sample P20_1000C_25% the overlap zone is not easily identifiable, and no significant microstructural changes are observed compared to that of the treated zone resulting from a single track.

**Fig. 7:**
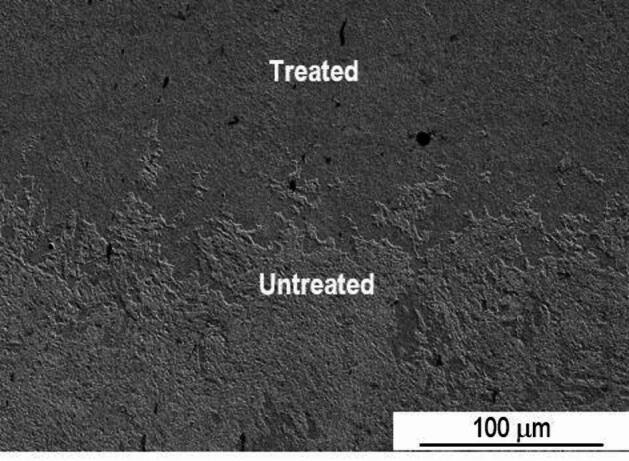
SEM SE image of the interface between the treated and untreated zone of sample P20_1200C_10%.

Due to the increase of temperature, the laser treated area of sample P20_1200C_10% is easily identifiable. Despite the overlap rate of 10%, in this case, using SEM, it is possible to distinguish the zone corresponding to the overlap of the two laser tracks. Compared to the zones subjected to a single passage of the laser beam, in the overlap zone no significant changes in the microstructure are observed. The interface between the treated and untreated zone is easy to distinguish, as illustrated in Fig. 7.

**Fig. 8:**
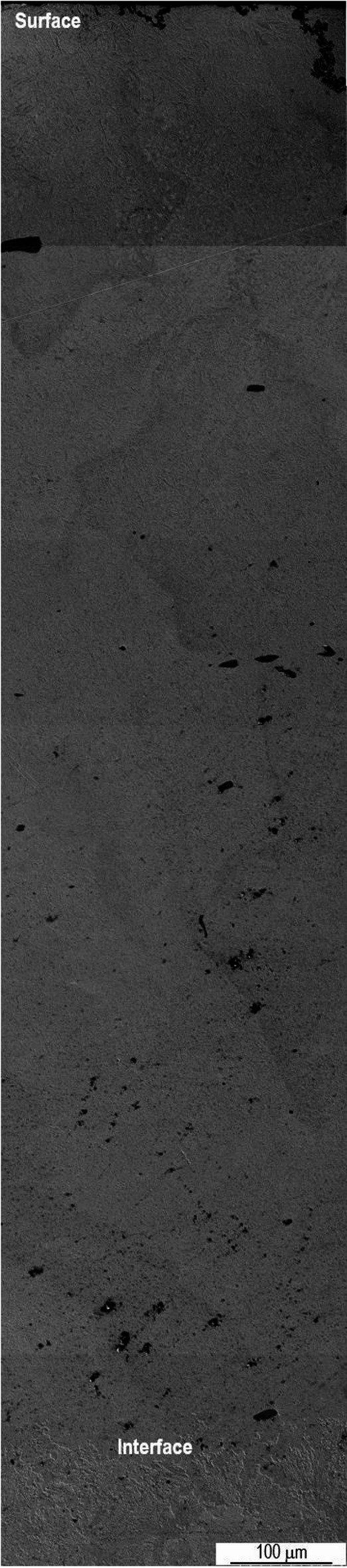
Reconstruction of the laser treated zone of sample P20_1200C_25%.

With the exception of the size of the overlap zone, sample P20_1200_25% does not present significant microstructural differences compared to sample P20_1200C_10%. Figure 8 shows the reconstruction of the area affected by the laser treatment of sample P20_1200C_25%. In this case, the laser track has a depth of approximately 1.4 mm.

**Fig. 9:**
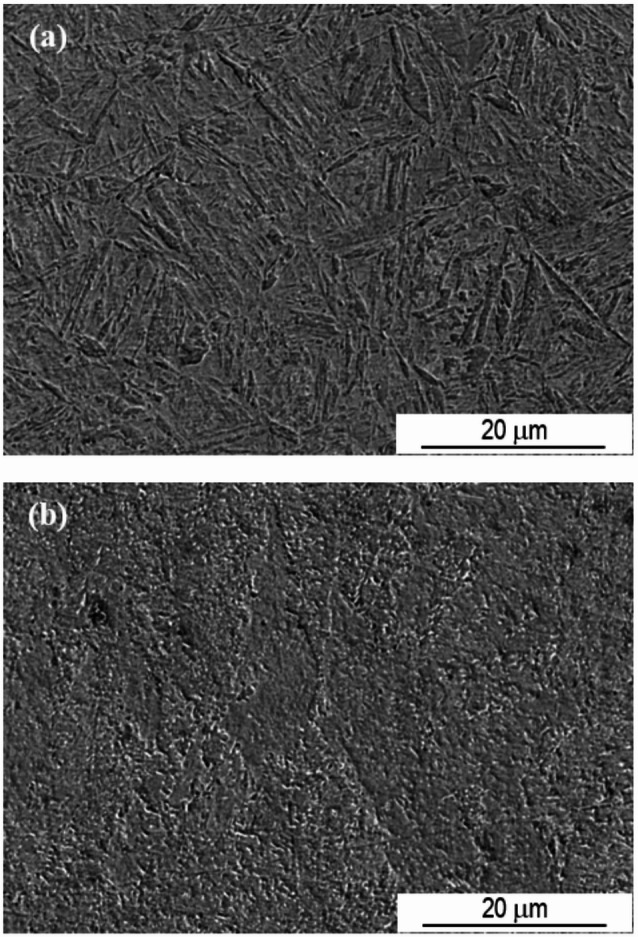
SEM SE images of sample P20_1200C_25%. (**a**) close to the treated surface; and (**b**) away from the laser affected zone.

Figure 9 allows the microstructure close to the surface of the laser-treated sample to be compared with that of the steel distant from the laser-affected zone. The microstructure near the quenched surface is distinct from that of the as-received steel (ferrite + pearlite), as already mentioned for the P20_1000C_25% sample. In relation to this sample, increasing the temperature to 1200 °C did not result in a significant change in the microstructure close to the quenched surface.

**Fig. 10:**
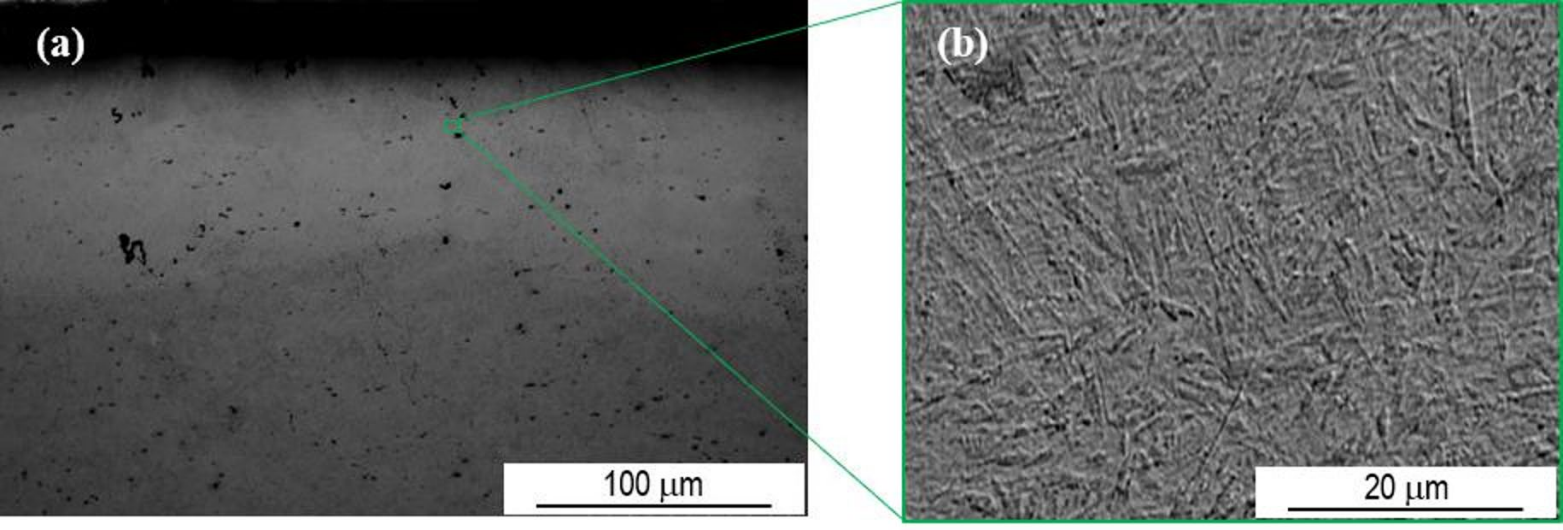
SEM images of the overlap zone of sample P20_1200C_25%. (**a**) BSE; (**b**) SE.

The zone corresponding to the overlap of the two laser marks of sample P20_1200C_25% can be identified, being more perceptible in BSE imaging (Fig. 10a). However, the microstructure does not present significant changes compared to that resulting from a single track of the laser beam (Fig. 10b versus Fig. 9a).

**Fig. 11:**
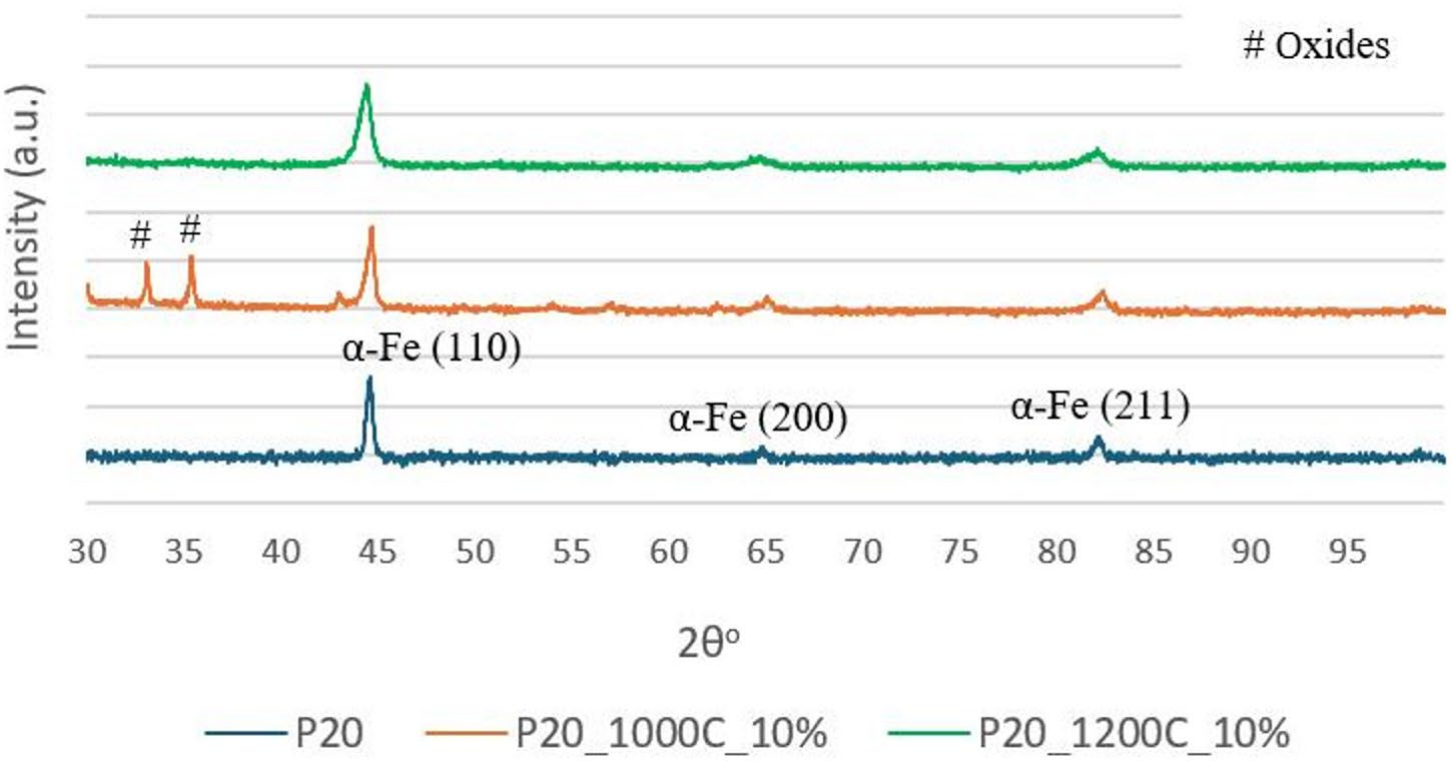
XRD diffractograms of laser-treated and as-received steel.

Figure 11 shows the X-ray diffractograms of laser-hardened P20 + S steel using heating temperatures of 1000 and 1200 °C, as well as of the as-received steel. The XRD results reveal that steel has a body centered cubic (BCC) structure, corresponding to the α-Fe phase. It is not possible by XRD to distinguish between BCC ferrite and body centered tetragonal martensite, and thus the presence of martensite cannot be confirmed. In the diffractogram of sample P20_1000C_10% it is possible to identify some additional X-ray peaks corresponding to iron oxides, namely hematite and magnetite. These peaks are not present in P20_1200C_10% because this sample was previously ground to remove the oxide layer.

#### Mechanical characterization

The laser quenched samples were subjected to nanoindentation tests with the aim of comparing the hardness before and after laser treatment, as well as evaluating the hardness variation along the cross-section of the treated steel samples.

**Fig. 12:**
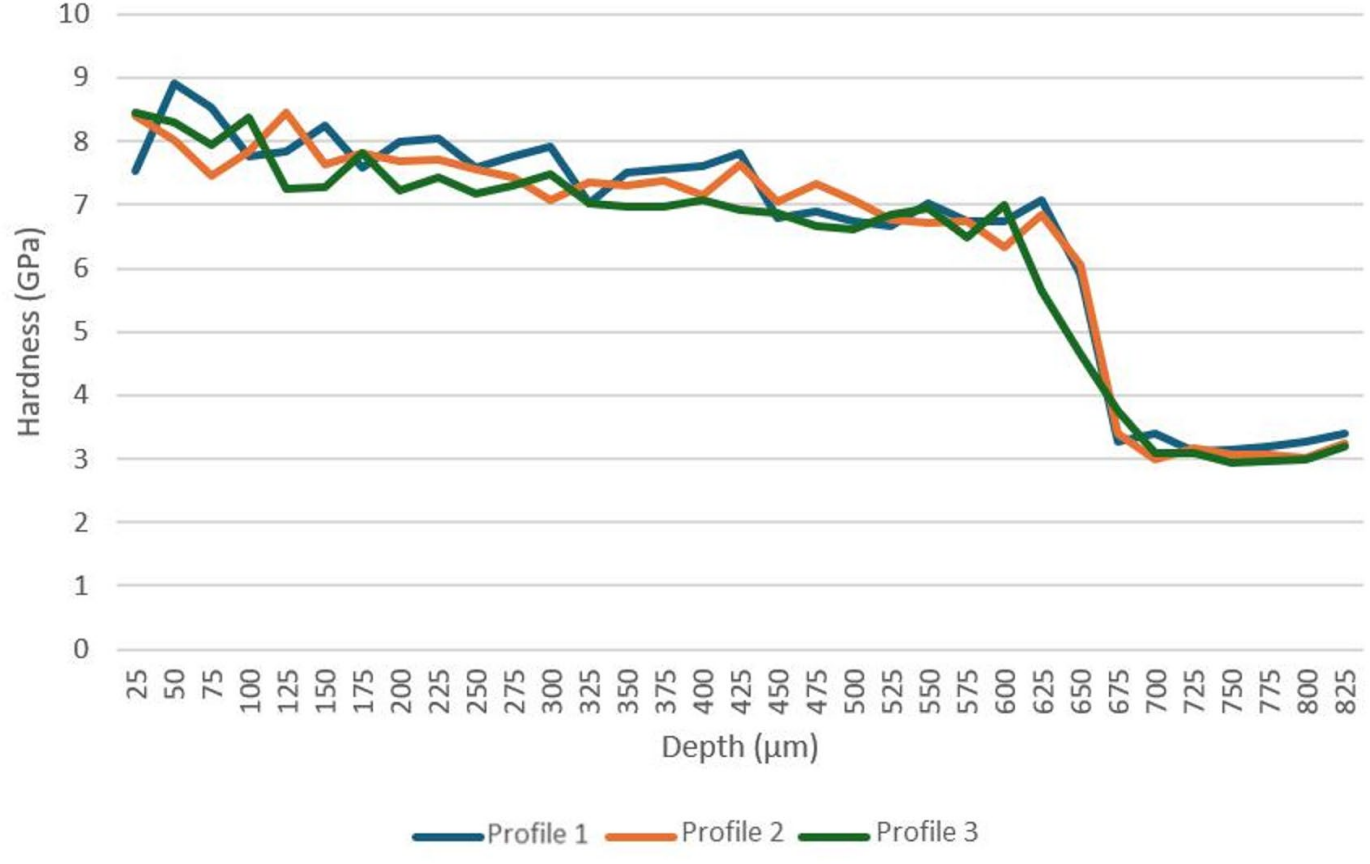
Hardness profiles of sample P20_1000C_25%.

Figure 12 shows three hardness depth profiles measured on P20 + S steel heated to 1000 °C and quenched. The results reveal that close to the surface and up to ∼400 μm depth the sample has a hardness above 7 GPa. This hardness value is consistent with the formation of martensite phase. In fact, although by XRD it is not possible to identify the presence of martensite, the hardness obtained can only be explained as resulting from the formation of this hard phase. Along sample P20_1000C_25% cross-section, the hardness slightly decreases, until an abrupt decrease occurs between 650 and 700 μm, reaching about 3 GPa for depths greater than 725 μm. This hardness is similar to the value presented for the untreated steel, indicating that the bulk of the sample remained unaltered. The depth from which the hardness of the untreated steel is reached is in accordance with the maximum depth of the treated zone measured by SEM.

The uniformity between the three profiles shown in Fig. 12 reinforces the effectiveness of surface hardening by laser treatment using a temperature of 1000 °C.

**Fig. 13:**
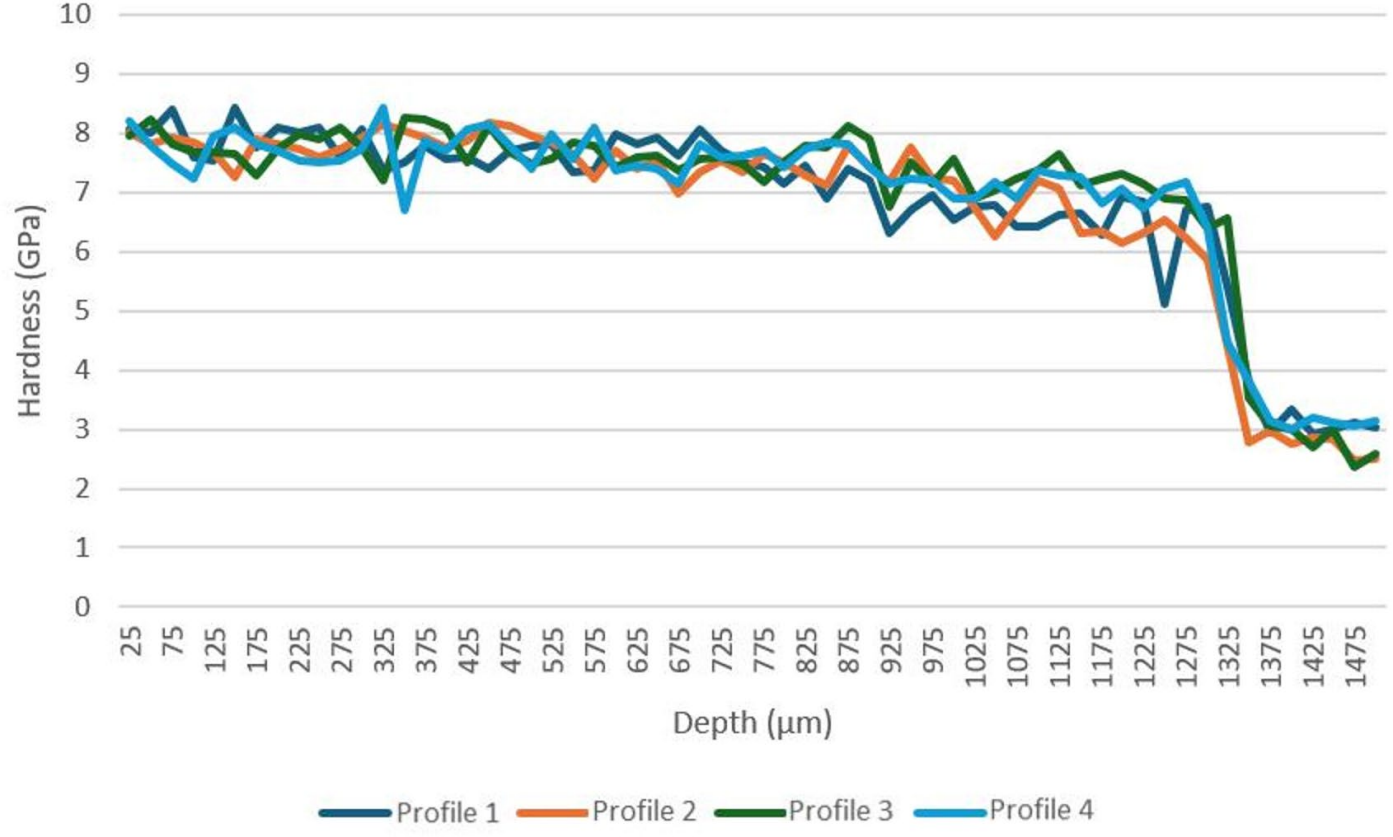
Hardness profiles of sample P20_1200C_10%.

Laser quenching using a temperature of 1200 °C was also effective, as demonstrated by the hardness profiles shown in Fig. 13. The four hardness profiles are similar, with a surface hardness of approximately 8 GPa. As expected, the increase of temperature from 1000 to 1200 °C, resulted in a significant increase of the depths presenting hardness values consistent with martensite. The increase of the quenching depth, has already been observed by OM and SEM. Below a depth of 1300 μm, the hardness decreases abruptly and reaches a value close to 3 GPa, corresponding to the hardness of the untreated steel.

The overlap rate between two laser tracks does not significantly alter the laser treatment effect in the deeper zones. In fact, the hardness profiles of sample P20_1200C_25% (not shown) are similar to those of sample P20_1200C_10%, although revealing slightly higher hardness and maximum depth.

**Fig. 14:**
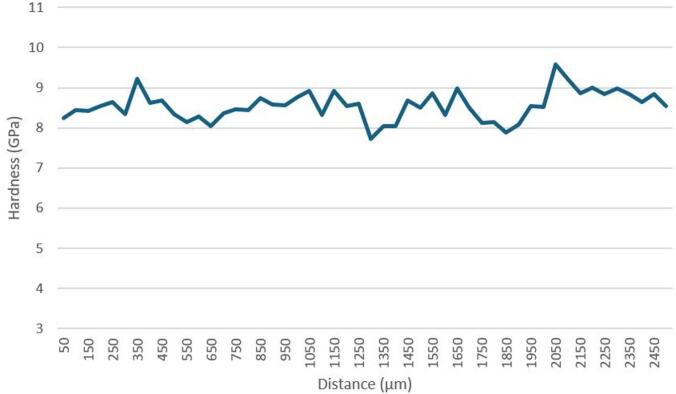
Hardness across overlap zone of sample P20_1200C_25%.

In sample P20_1200C_25%, the zone corresponding to the overlap of the two laser tracks is clearly visible, which allowed nanoindentation tests to be carried out across this zone (see Fig. 1). Analyzing the hardness across the entire width of the overlap zone (Fig. 14), it can be seen that there are no significant hardness variations. In the overlap zone, the hardness is identical to that observed near the surface in the zone corresponding to the maximum depth, thus away from the overlap region. The hardness values are in line with expectations, considering the absence of significant microstructural changes in the overlap zone.

**Fig. 15:**
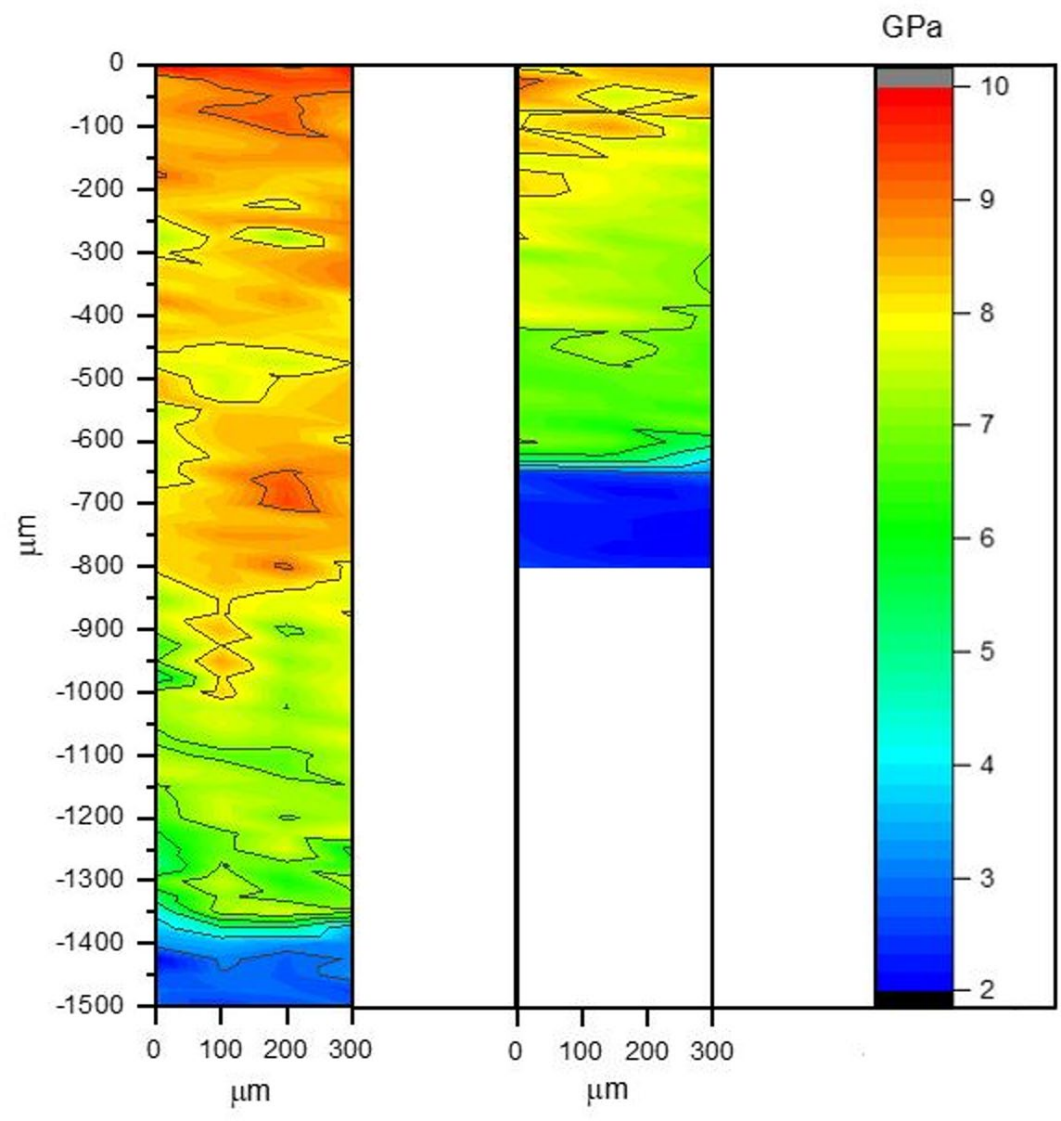
Hardness maps of samples P20_1200C_25% (left) and P20_1000C_25% (right).

The hardness maps in Fig. 15 allow the effect of heating temperature on the hardness of laser-treated samples to be easily observed. Both samples have similar hardness near the surface. The top surface hardness (average of the four indentations closest to the surface for each profile) of samples P20_1000_25% (8.1 ± 0.42 GPa) and P20_1200_25% (8.9 ± 0.4 GPa) is significantly higher that the hardness of the as-received steel (3.4 ± 0.22 GPa). However, the maps are distinct, highlighting the significant difference in the maximum depth of the treated zone; close to 650 and 1400 μm for heating temperatures of 1000 and 1200 °C, respectively. In addition, for sample P20_1200C_25% it is possible to observe in Fig. 15 that the maximum hardness is maintained up to depths of 800–850 μm, while in sample P20_1000C_25% the maximum hardness is limited to the surface (below intermediate hardness values are observed).

#### Tribological behaviour

**Fig. 16:**
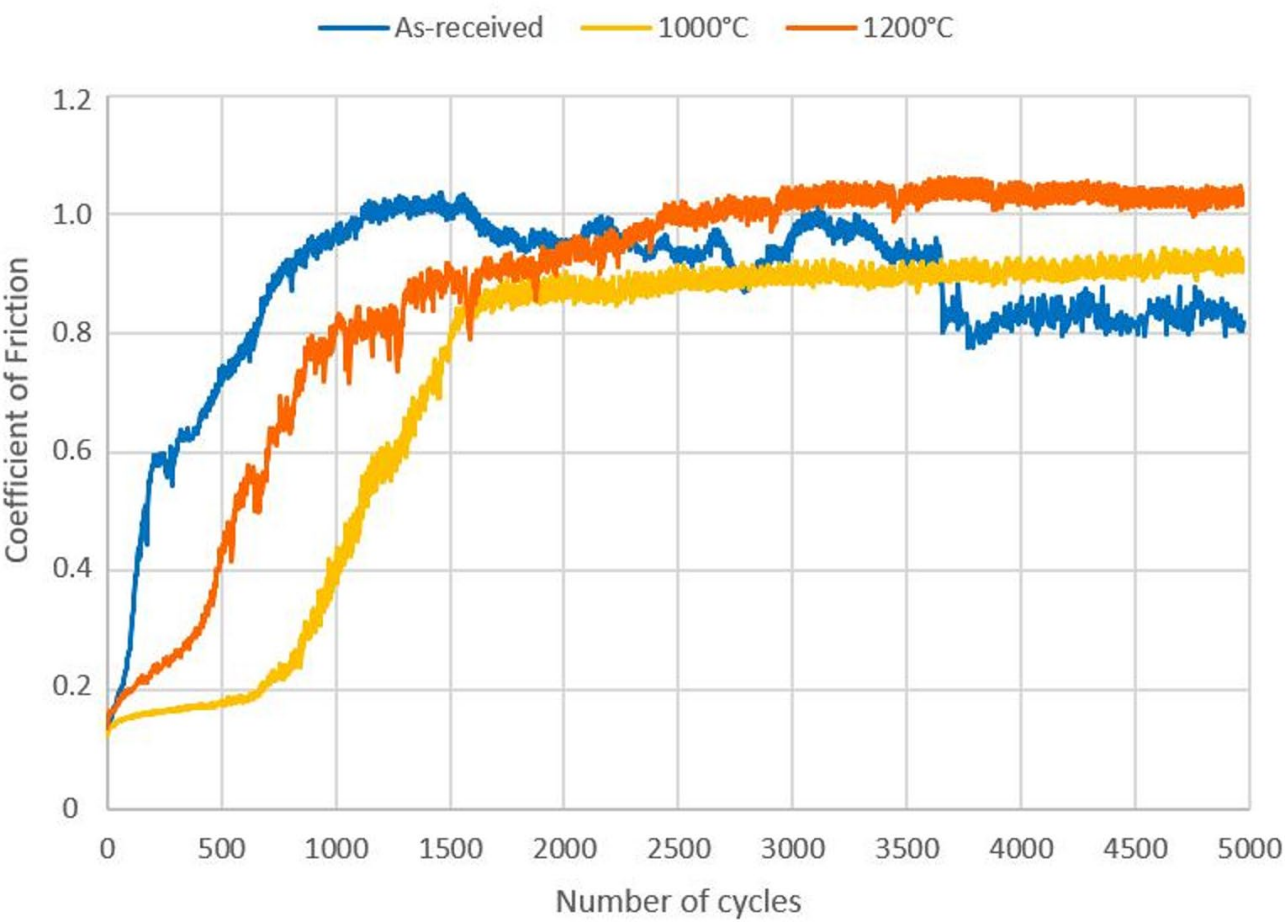
Coefficient of friction of as-received and treated steel.

The evolution of the coefficient of friction (CoF) with the number of cycles is represented in Fig. 16. For comparison purposes the as-received steel is also included, whose CoF increases up to 1 and, after ∼ 3800 cycles, stabilizes slightly above 0.8. The CoF of the laser hardened steel using a temperature of 1000 °C stabilizes at 0.9 and, comparing with the as-received steel, presents a more stable profile and rather smooth behaviour during the 5000 cycles.

The laser quenching using a temperature of 1200 °C results in a more irregular behaviour and the CoF stabilizes close to 1.0. The profile is significantly wider and steeper than that observed for the as-received steel and treated using a temperature of 1000 °C. These results suggest a different wear mechanism at higher temperatures. It should be noticed, that the samples laser hardened using a temperature of 1200 °C show clear signs of oxidation, which influences the tribological behaviour and explains the values of the wear rate and volume in Table 2.

**Table 2:** Specific wear rate (*k*) and wear volume of the as-received and laser treated steel.

	K (mm^3^/*N*.m)	V (mm^3^)
P20 + S	1.72 × 10^− 5^	2.70 × 10^− 2^
P20_1000C_25%	2.75 × 10^− 5^	4.31 × 10^− 2^
P20_1200C_25%	9.74 × 10^− 5^	1.53 × 10^− 1^

The laser treated steel, in particular sample P20_1200C_25%, exhibits significantly higher wear rate and volume. In fact, although surface hardening was effective, oxidation may have compromised the wear behaviour.

**Fig. 17:**
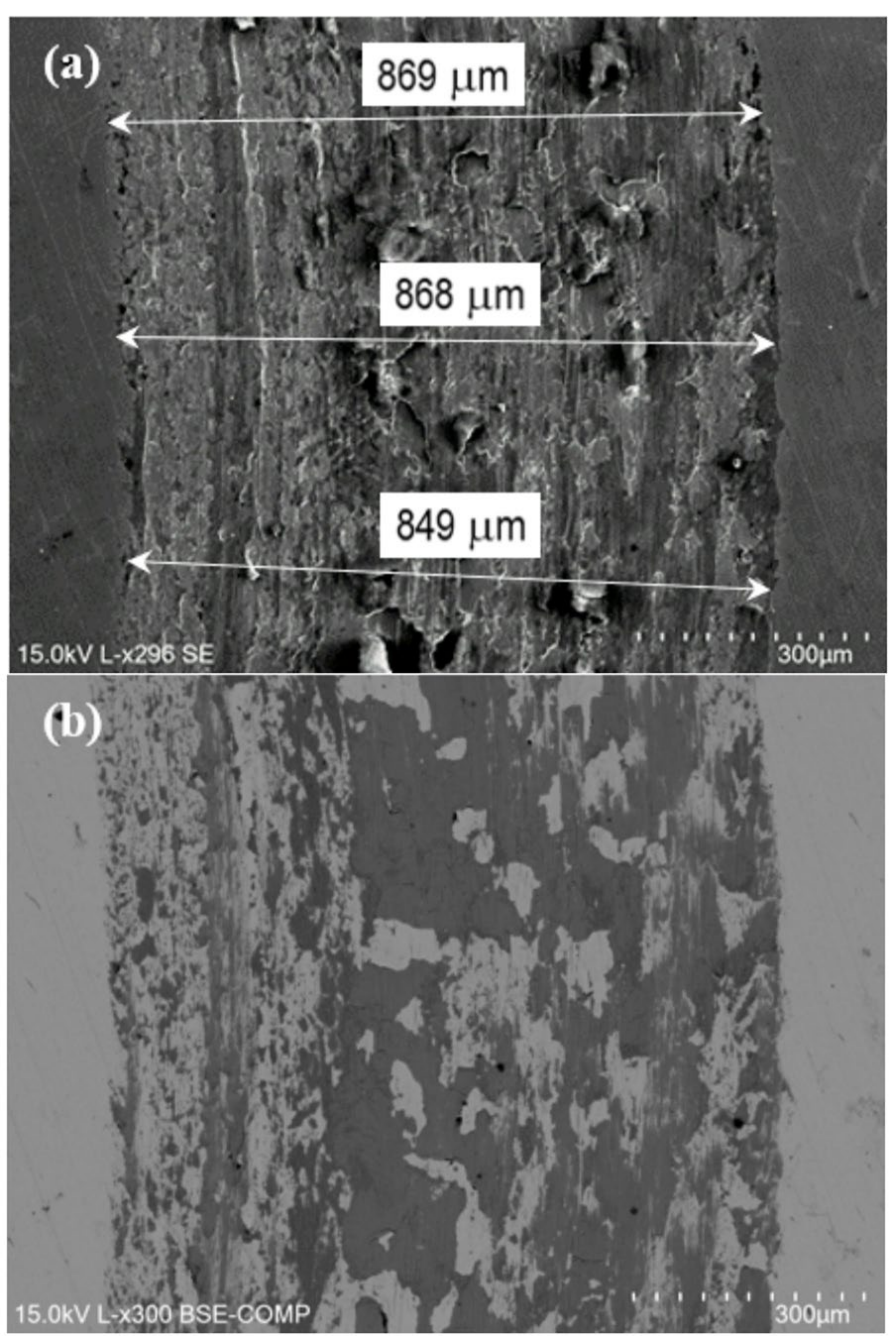
SEM images of a P20 + S steel wear track. (**a**) SE, and (**b**) BSE.

**Fig. 18:**
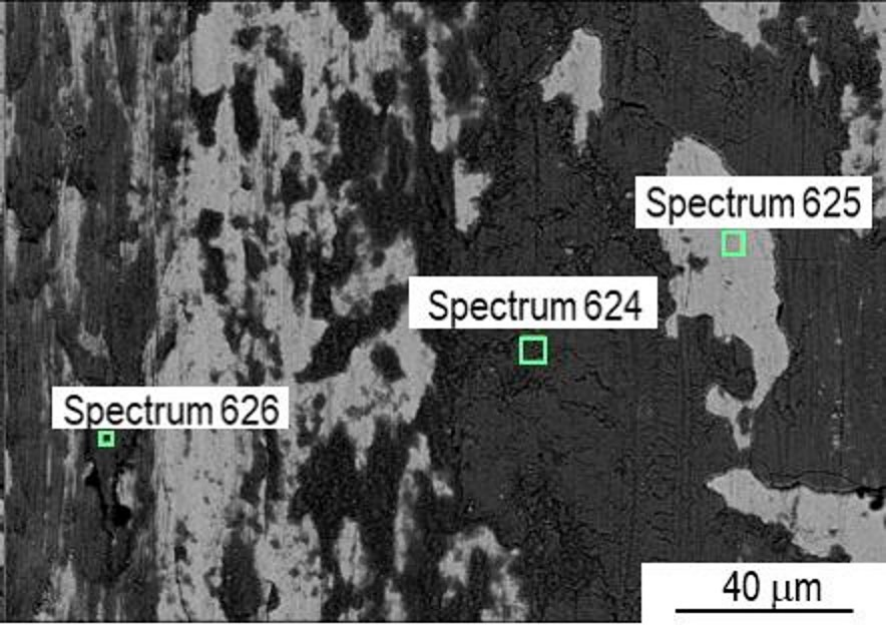
SEM image of a P20 + S steel wear track with EDS areas pointed out.

In order to examine the wear mechanism, the wear tracks were analysed by SEM/EDS. The P20 + S steel presents a homogeneous wear track, with a few abrasion risks and fish-like type wear debris, as observed in Fig. 17. EDS analysis in the darker area of the track (spectrum 624, Fig. 18), corresponding to the oxidized layer, revealed a significantly higher oxygen content, as well as a higher silicon content of 5.4 wt% Si (Figure S1). The increase in the Si content should be related to the wear of the Si_3_N_4_ counterbody.

**Fig. 19:**
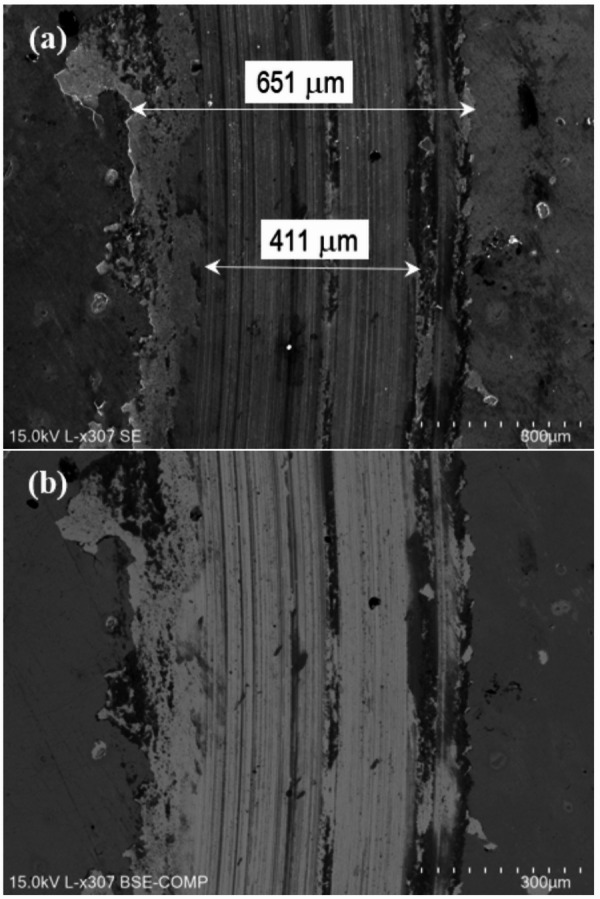
SEM images of a P20_1000C_25% wear track. (**a**) SE, and (**b**) BSE.

**Fig. 20:**
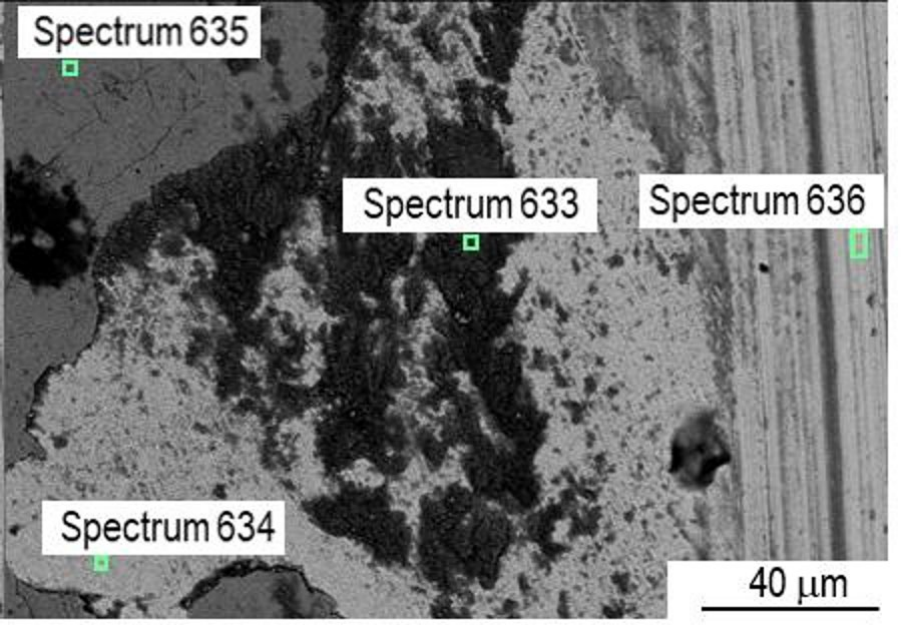
SEM image of a P20_1000C_25% wear track with EDS areas pointed out.

In sample P20_1000C_25%, material accumulates at the edges of the wear track as can be observed in Fig. 19. This is due to the displacement of the oxide layer formed during the test outside the track, making it difficult to measure track’s width. During the wear test, the oxide layer resulting from laser hardening detached. This oxide layer has poor adhesion, and outside the wear tracks there are some regions where the oxide layer detached, explaining the higher wear coefficient and volume observed compared with the as-received steel (Table 2). The abrasion marks are more significant than in the untreated steel (Fig. 17*versus* Fig. 19), indicating a different wear mechanism. EDS analysis is particularly useful in this sample (Fig. 20), as it allows the oxide layer resulting from laser hardening and the oxide layer formed during the pin-on-disk test to be distinguished. Indeed, in both cases the oxygen content is significant, but, while in the oxidized layer outside the track (spectrum 635, Fig. 20) there is no silicon (Figure S4), due to the counterbody material the Si content increases to close to 10 wt% (Figure S3) in the oxide formed during the wear test (spectrum 633, Fig. 20).

**Fig. 21:**
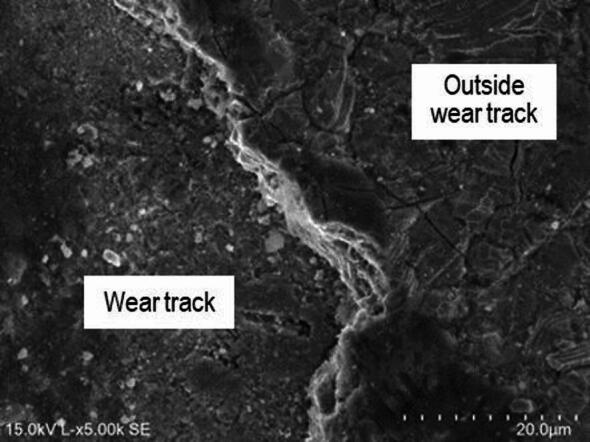
SEM image of the border of a P20_1200C_25% wear track.

Sample P20_1200C_25% has a different tribological behaviour, due to the thicker oxide layer formed on the surface. The oxide layer resulting from laser hardening appears to have been torn off during the test, with subsequent exposure of the hardened surface (Fig. 21), which explains the abrupt and wider profile observed.

**Fig. 22:**
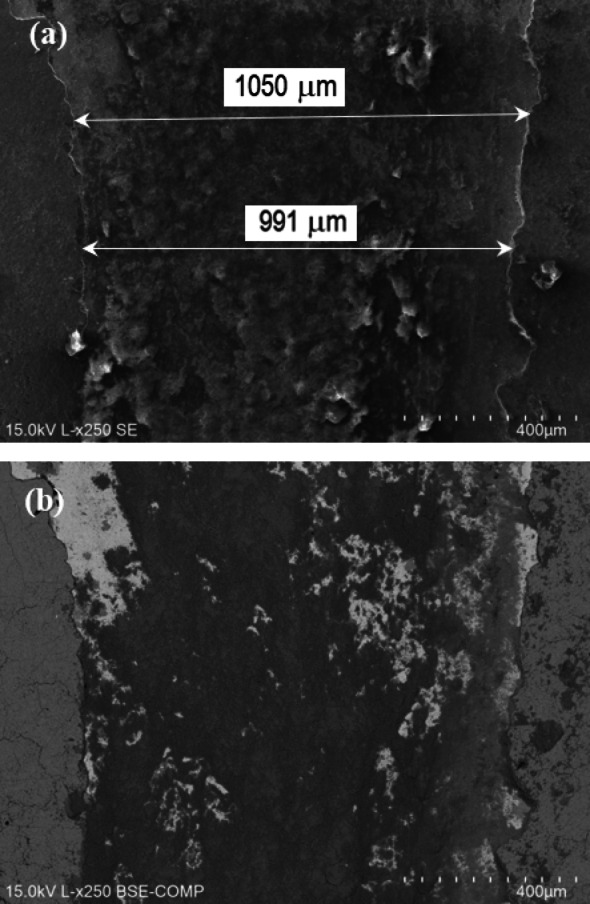
SEM images of a P20_1200C_25% wear track. (**a**) SE, and (**b**) BSE.

**Fig. 23:**
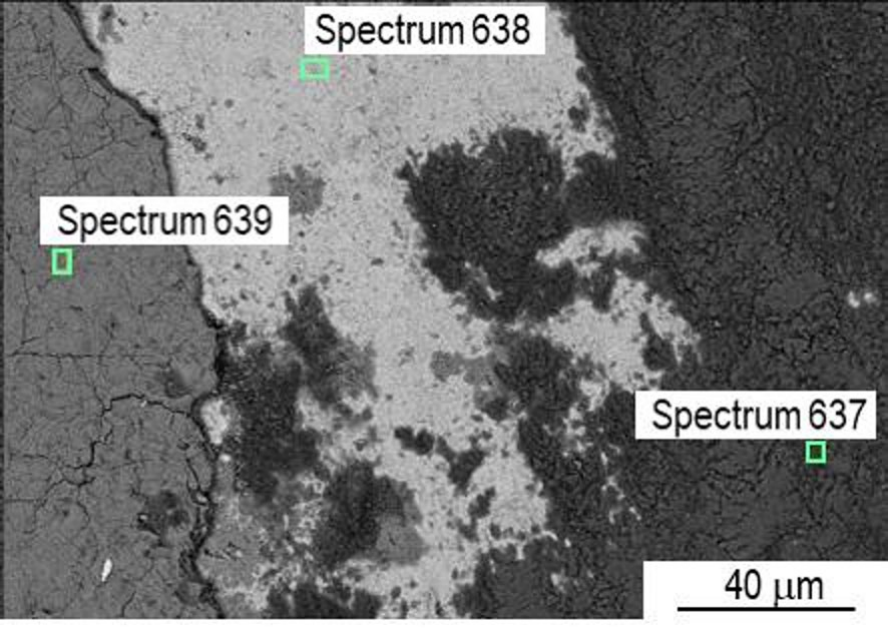
SEM image of a P20_1200C_25% wear track with EDS areas pointed out.

The wear track of P20_1200C_25% sample (Fig. 22) is notably wider, suggesting that the Si_3_N_4_ sphere is more worn, as expected, since this sample is harder. During the pin-on-disk test, an oxide layer is formed that is successively removed and formed again—adhesion wear. Again, the EDS analysis (Fig. 23) allows the distinction between the oxide layer resulting from laser hardening (spectrum 639, Fig. 23) and the oxide formed during the wear test (spectrum 637, Fig. 23), the latter having a silicon content of ∼11 wt% due to the incorporation of material from the counterbody (Figure S5). In the laser-induced oxide, silicon is not detected by EDS (Figure S7). The chemical composition in the exposed zone (spectrum 638, Fig. 23) corresponds to the nominal composition of the steel (Figure S6).

The dominant wear mechanism observed was adhesive wear, associated with the detachment of oxide layers formed during laser treatment and during the pin-on-disk tests. Localized frictional heating and harsh increase in the flash temperature during sliding, even for room temperature wear tests, can promote tribo-oxidation, leading to the formation and removal of oxide debris. Detached oxide fragments and transferred material from the Si₃N₄ counterbody, evidenced by increased silicon content in the wear tracks, may act as third-body particles, introducing mild abrasive effects, as observed for the 1000 °C sample (Fig. 19).

Since the oxide layer on P20_1000C_25% is much thinner than on P20_1200C_25%, for the 1000 °C sample the wear performance was not significantly affected by the laser-induced oxide layer. When minor detachment occurred, the underlying martensitic layer continued to provide effective wear resistance. In contrast, the sample treated at 1200 °C developed a thicker oxide layer with poor adhesion, which detached during sliding, exposing and damaging the surface repeatedly.

##  Conclusion

This work allowed a detailed analysis of the microstructural characteristics, hardness and wear behaviour of P20 + S mould steel surface hardened by laser quenching, using different temperatures and overlap rates of the laser tracks.

Laser quenching using a temperature of 1000 °C resulted in a depth slightly lower than 700 μm and a surface hardness close to 8 GPa. The hardness increase compared to the as-received steel is attributed to the formation of martensite. As the temperature increased to 1200 °C, the depth of the quenched zone increased to near 1400 μm, and the surface hardness reached 8 to 9 GPa. In the area corresponding to the overlap of the laser beam tracks, no significant microstructural changes or hardness variations were detected. The increase of temperature promoted the formation of a thicker oxide layer on the laser treated surface, which influenced the wear behaviour. In fact, at 1200 °C the steel showed a significant increase of the wear volume. This increase is attributed to the detachment of the oxide layer formed during the laser treatment. The dominant mechanism observed was adhesive wear, associated with the detachment of oxide layers. Since the oxide layer formed at 1000 °C is much thinner than for 1200 °C, the wear performance of P20_1000C_25% was not significantly affected by the laser-induced oxide layer.

In conclusion, laser quenching is a fast and effective alternative for surface hardening of mould steels.

## Supplementary Information

Below is the link to the electronic supplementary material.

Supplementary Material 1

## Data Availability

Data generated during the current study are available from the corresponding author on reasonable request. The laser quenching process data are available from Durit Coatings company, but restrictions apply to the availability of these data due to confidentiality.

## References

[1] Surface hardening of steels: Understanding the basics. J.R. Davis (Ed.). ASM International, Ohio. ISBN: 0-87170-764-0, 978-0-87170-764-2. (2002).

[2] Afanas’eva, L. E., Novoselova, M. V., Barabonova, I. A. & Ratkevich, G. V. Effect of laser quenching on the microstructure and the abrasive wear resistance of 30KhGSA steel. *Russ Metall.* **2020**, 45–49. 10.1134/S0036029520010024 (2020).

[3] Fischer, A., Scholtes, B. & Niendorf, T. On the Influence of surface hardening treatments on microstructure evolution and residual stress in microalloyed medium carbon steel. *J. Mater. Eng. Perf*. **29**, 3040–3054. 10.1007/s11665-020-04866-y (2020).

[4] Lin, Y. C., Wang, S. W. & Chen, T. M. A study on the wear behavior of hardened medium carbon steel. *J. Mater. Process. Technol.* **120**, 126–132. 10.1016/S0924-0136(01)01195-5 (2002).

[5] Wang, L. Y. et al. Strain hardening behaviour of as-quenched and tempered martensite. *Acta Mater.* **199**, 613–632. 10.1016/j.actamat.2020.08.067 (2020).

[6] Samuel, A., & Narayan Prabhu, K. Residual stress and distortion during quench hardening of steels: A review. *J. Mater. Eng. Perf*. **31**, 5161–5188. 10.1007/s11665-022-06667-x (2022).

[7] Ion, J. C. Laser transformation hardening. *Surf. Eng.* **18**, 14–31. 10.1179/026708401225001228 (2002).

[8] Pantelis, D. I., Bouyiouri, E., Kouloumbi, N., Vassiliou, P. & Koutsomichalis, A. Wear and corrosion resistance of laser surface hardened structural steel. *Surf. Coat. Technol.* **161**, 125–134. 10.1016/S0257-8972(02)00495-4 (2002).

[9] El-Batahgy, A., Ramadan, R. A. & Moussa, A. Laser surface hardening of tool steels-experimental and numerical analysis. *J. Surf. Eng. Mater. Adv. Technol.* **3**, 146–153. 10.4236/jsemat.2013.32019 (2013).

[10] Chun, E. J., Sim, A., Kim, M. S. & Kang, N. Microstructural characterization of surface softening behavior for Cu-bearing martensitic steels after laser surface heat treatment. *Metals* **8**, 470. 10.3390/met8060470 (2018).

[11] Shin, W. S. et al. Effect of laser heat-treatment and laser nitriding on the microstructural evolutions and wear behaviors of AISI P21 mold steel. *Metals* **10**, 1487. 10.3390/met10111487 (2020).

[12] Lagarinhos, J. N., Afonso, D., Torcato, R., Santos, C. & Oliveira, M. Effect of laser heat treatments on the hardness of tool steels. *IOP Conf. Ser. Mater. Sci. Eng.* **1193**, 012026. 10.1088/1757-899X/1193/1/012026 (2021).

[13] Goia, F. A. & de Lima, M. S. F. Surface hardening of an AISI D6 cold work steel using a fiber laser. *J. ASTM Int.* **8**, 1–9. 10.1520/JAI103210 (2011).

[14] Telasang, G., Dutta Majumdar, J., Padmanabham, G. & Manna, I. Structure–property correlation in laser surface treated AISI H13 tool steel for improved mechanical properties. *Mater. Sci. Eng. A*. **599**, 255–267. 10.1016/J.MSEA.2014.01.083 (2014).

[15] Cordovilla, F. et al. Numerical/experimental analysis of the laser surface hardening with overlapped tracks to design the configuration of the process for Cr–Mo steels. *Mater. Des.* **102**, 225–237. 10.1016/j.matdes.2016.04.038 (2016).

[16] Moradi, M., Arabi, H., Jamshidi Nasab, S. & Benyounis, K. Y. A comparative study of laser surface hardening of AISI 410 and 420 martensitic stainless steels by using diode laser. *Opt. Laser Technol.* **111**, 347–357. 10.1016/j.optlastec.2018.10.013 (2019).

[17] Park, C. et al. Influence of high-power diode laser heat treatment on wear resistance of a mold steel. *J. Mec Sci. Technol.* **33**, 829–836. 10.1007/s12206-019-0139-y (2019).

[18] Park, C., Sim, A., Ahn, S., Kang, H. & Chun, E. J. Influence of laser surface engineering of AISI P20-improved mold steel on wear and corrosion behaviors. *Surf. Coat. Technol.* **377**, 124852. 10.1016/J.SURFCOAT.2019.08.006 (2019).

[19] Oliver, W. C. & Pharr, G. M. Measurement of hardness and elastic modulus by instrumented indentation: Advances in understanding and refinements to methodology. *J. Mater. Res.* **19**, 3–20. 10.1557/jmr.2004.0002 (2004).

[20] ASTM-G99-. 95 - Standard Test Method for Wear Testing with a Pin-on-Disk Apparatus.

[21] Holmberg, K., Matthews, A. & Ronkainen, H. Coatings tribology: Contact mechanisms and surface design. *Tribol. Int.* **31**, 107–120. 10.1016/S0301-679X(98)00013-9 (1998).

