# Supplementary material for: Surface hardening of a mould steel by laser quenching
Source: Sci Rep. 2026 Mar 10;16:12917. doi: 10.1038/s41598-026-42194-8 (PMC13096205; doi:10.1038/s41598-026-42194-8)
Supplement: Supplementary file 1 — Supplementary Material 1 [file 41598_2026_42194_MOESM1_ESM.docx]

Supplementary Material

EDS spectra (Figure 18, 20 and 23).


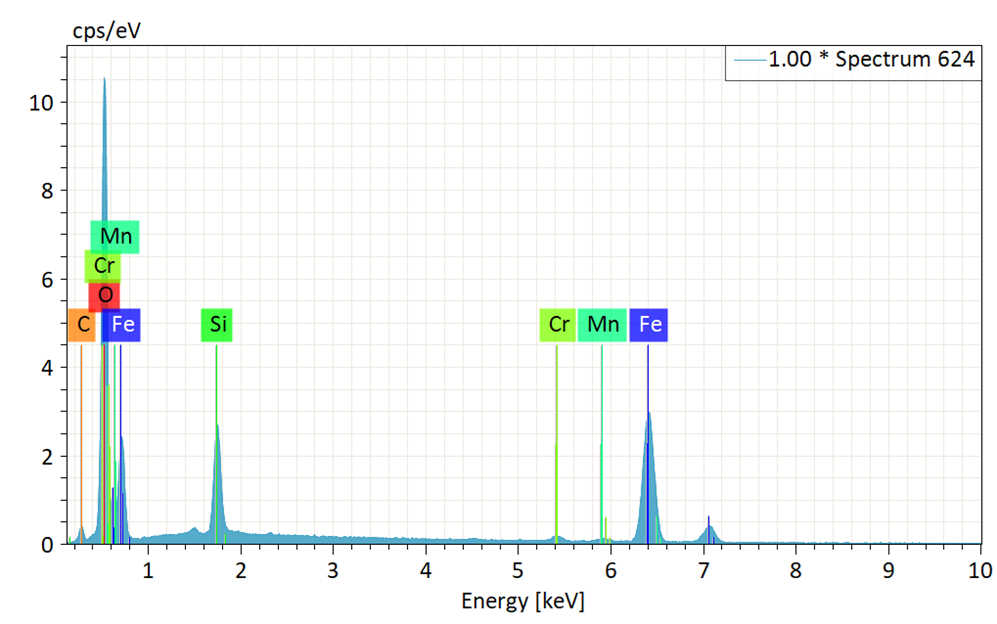


Figure S1 – EDS spectrum 624 (Figure 18).


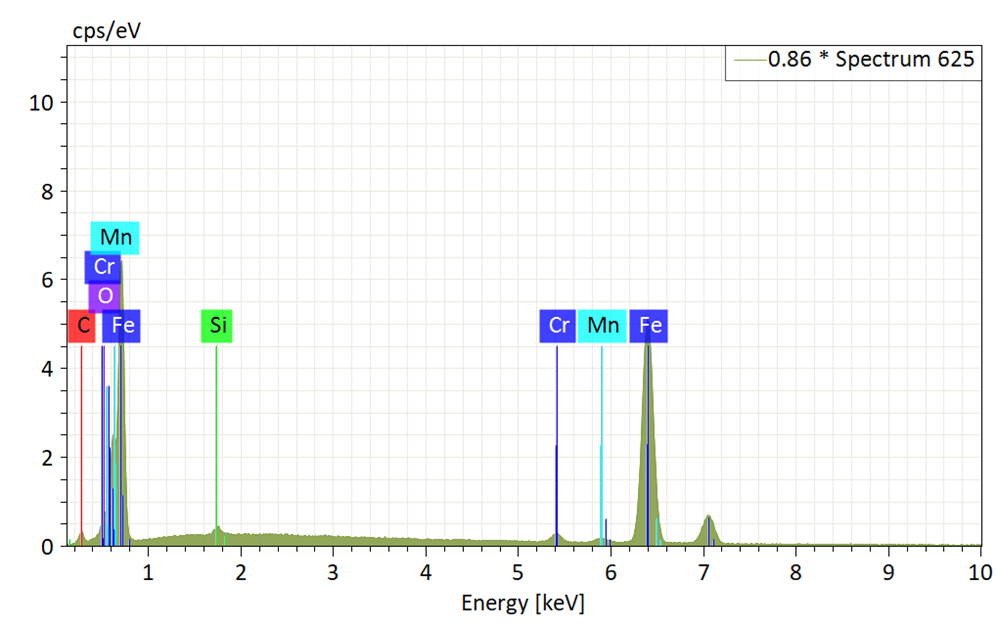


Figure S2 – EDS spectrum 625 (Figure 18).


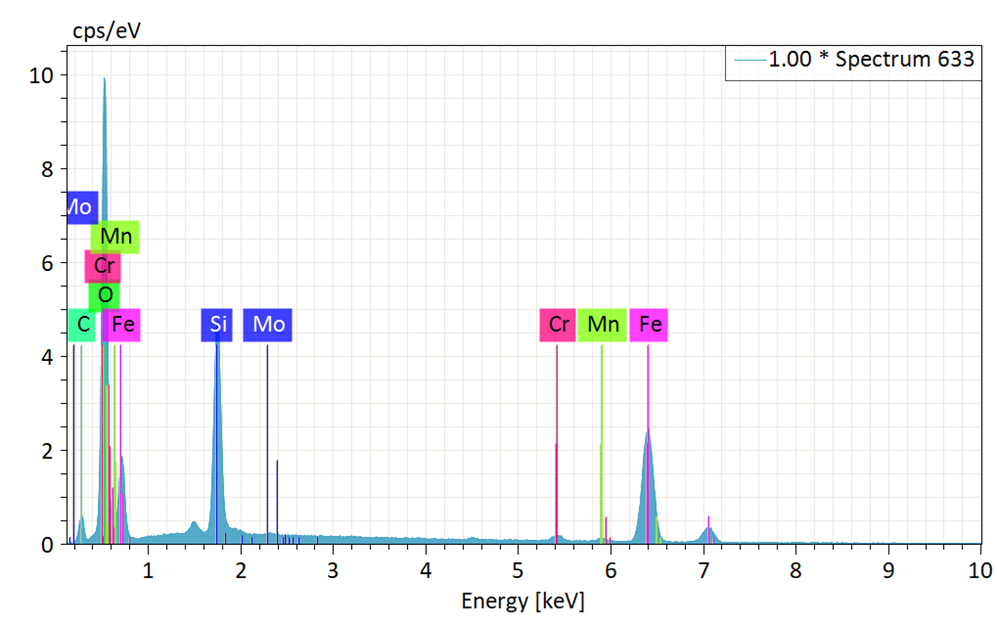


Figure S3 – EDS spectrum 633 (Figure 20).


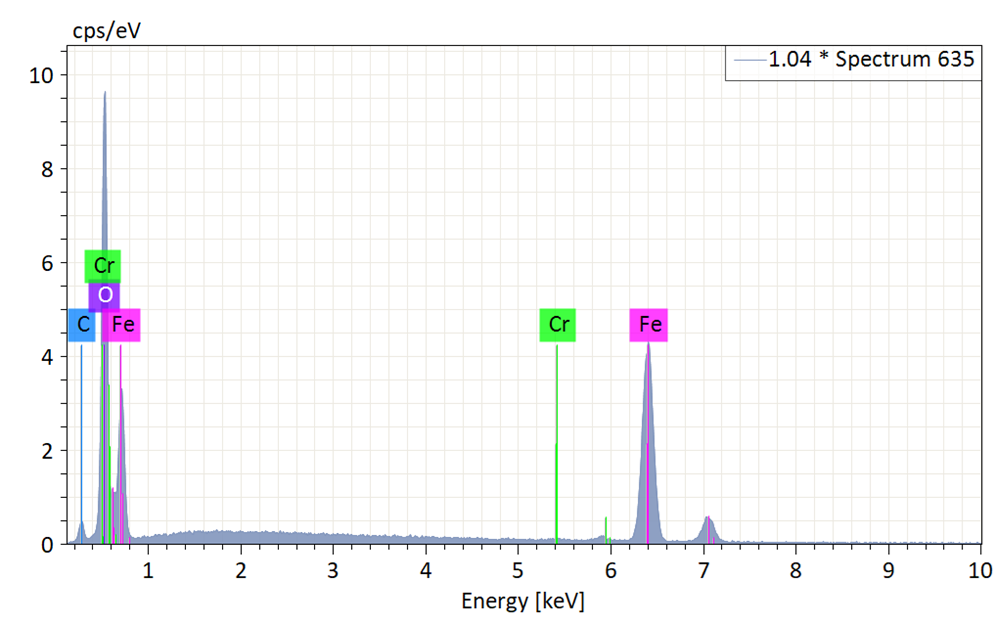


Figure S4 – EDS spectrum 635 (Figure 20).


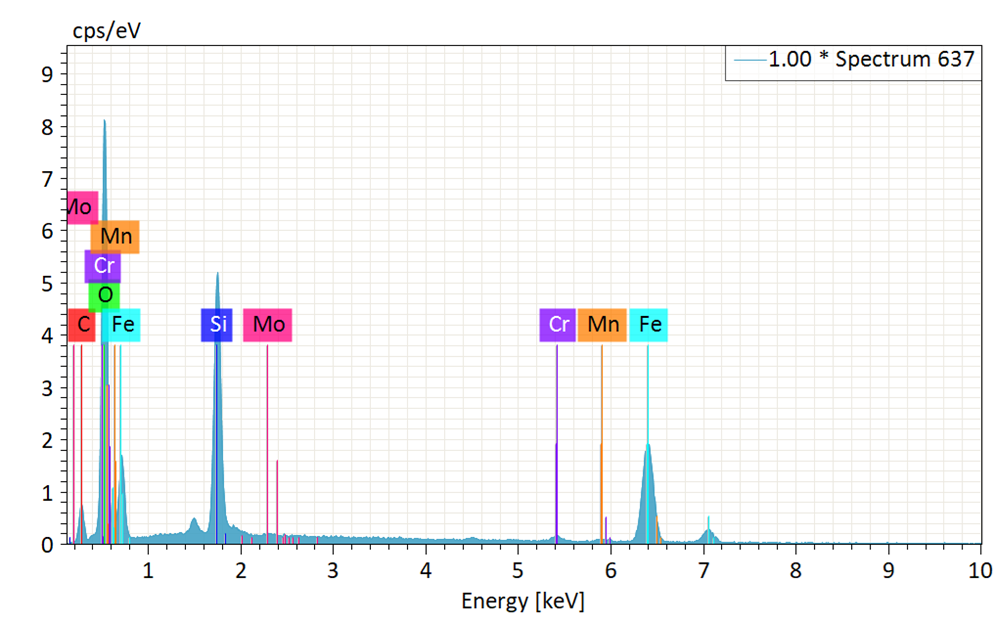


Figure S5 – EDS spectrum 637 (Figure 23).


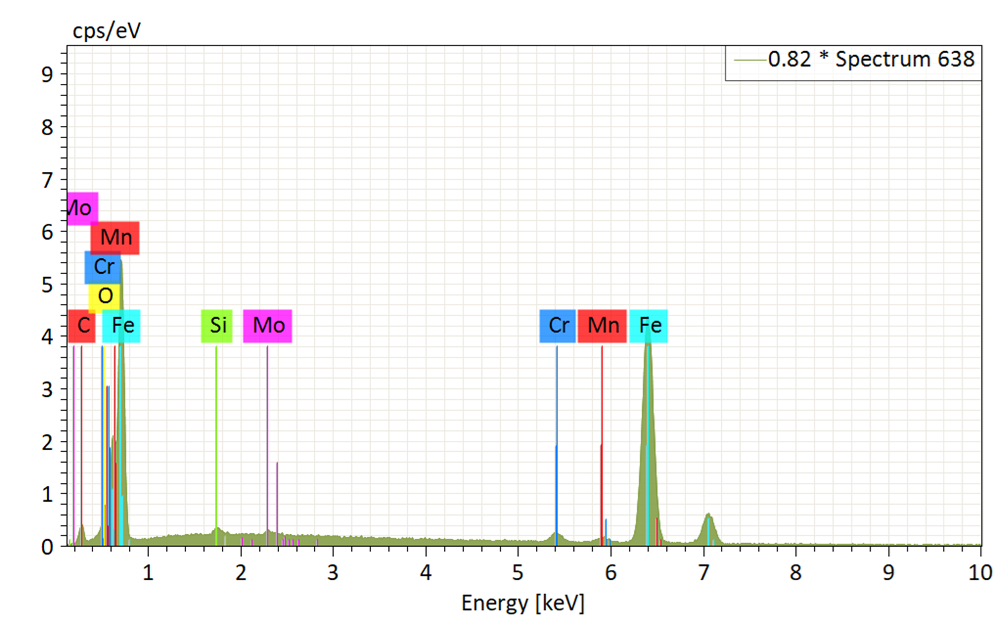


Figure S6 – EDS spectrum 638 (Figure 23).


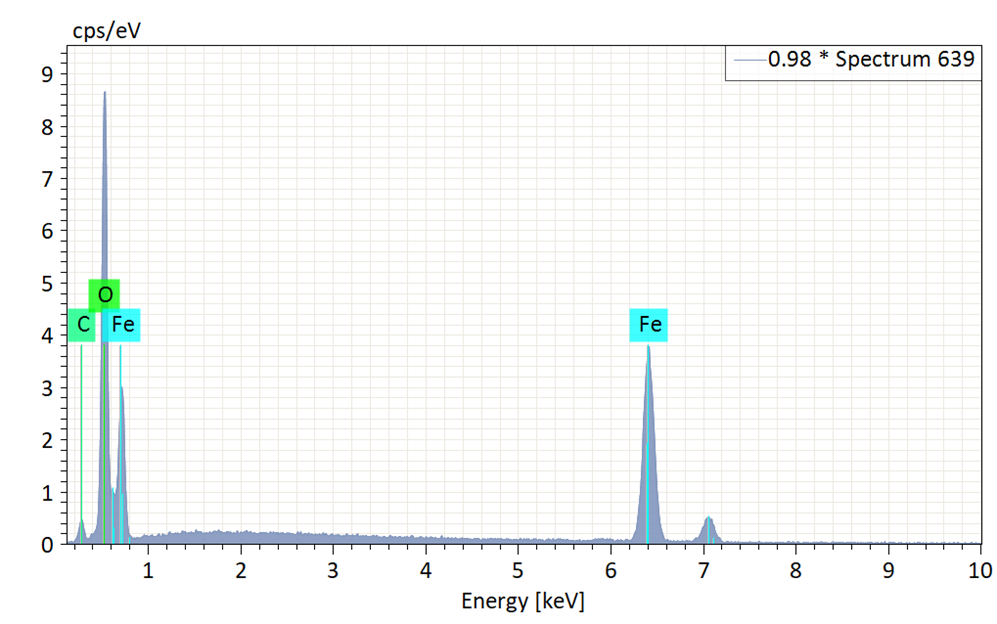


Figure S7 – EDS spectrum 639 (Figure 23).
